# Alexander Technique vs. Targeted Exercise for Neck Pain—A Preliminary Comparison

**DOI:** 10.3390/app11104640

**Published:** 2021-05-19

**Authors:** Jordan J. Becker, Tara L. McIsaac, Shawn L. Copeland, Rajal G. Cohen

**Affiliations:** 1 Department of Psychology & Communication, University of Idaho, Moscow, ID 83844, USA;; 2 National Center of Complementary and Integrative Health, National Institutes of Health, Bethesda, MD 20892, USA; 3 Department of Physical Therapy, A.T. Still University, Mesa, AZ 85206, USA;; 4 Lionel Hampton School of Music, University of Idaho, Moscow, ID 83844, USA;

**Keywords:** rehabilitation, posture, exercise, cranio-cervical flexion test, CCFT, electromyography, self-efficacy, muscle fatigue, self-care, integrative medicine

## Abstract

**Background:**

Alexander technique private lessons have been shown to reduce chronic neck pain and are thought to work by different mechanisms than exercise. Group classes may also be effective and would be cost-effective.

**Design:**

A two-group pre-test/post-test design. Participants were assigned to either a general Alexander technique class or an exercise class designed to target neck pain. Both groups met over 5 weeks for two 60 min sessions/week.

**Participants:**

A total of 16 participants with chronic neck pain (aged 50+/−16 years) completed this study.

**Interventions:**

The Alexander class used awareness-building methods to teach participants to reduce habitual tension during everyday activities. The exercise class was based on physical therapy standard of care to strengthen neck and back muscles thought to be important for posture.

**Measures:**

We assessed neck pain/disability, pain self-efficacy, activation of the sternocleidomastoid muscles during the cranio-cervical flexion test, and posture while participants played a video game.

**Results:**

Both groups reported decreased neck pain/disability after the interventions. Sternocleidomastoid activation decreased only in the Alexander group.

**Conclusion:**

In this small preliminary study, Alexander classes were at least as effective as exercise classes in reducing neck pain and seemed to work via a different mechanism. Larger, multi-site studies are justified.

## 1. Introduction

With an annual prevalence of approximately 26% [1], neck pain is a leading cause of disability in the US [2]. Persistent neck pain and dysfunction can secondarily cause disabling headaches and contribute to lost productivity. The burden of neck and back pain is staggering, accounting for $87.6 billion of estimated health care spending in the U.S. and 35% of the lost workdays in 2013 [3,4]. People with chronic neck pain can demonstrate maladaptive pain cognitions, including psychological distress, anxiety, depression, pain catastrophizing, and fear avoidance [5]. Potential causes of neck pain include pain self-efficacy, faulty postural alignment, and inefficient use of the postural muscles. According to the biopsychosocial model, chronic pain is influenced by self-efficacy, which has to do with a person’s beliefs about their ability to cope with pain and the negative emotions associated with pain, and with their confidence that they can carry out their daily activities under adverse conditions [6]. Pain self-efficacy may be an important link between pain and disability [7,8]. Another factor that has been proposed to contribute to neck pain is postural alignment. Evidence for this comes from studies showing that people with neck pain tend to habitually carry their necks forward from their spines [9] (forward neck posture) and that forward neck posture increases muscle activation in the superficial neck flexors [10–12]. Altered coordination of neck muscles may also contribute to neck pain. This is indicated by studies demonstrating that people with neck pain are more likely than those without neck pain to activate the superficial sternocleidomastoid muscles, rather than the deeper cervical muscles, during the cranio-cervical flexion test (CCFT), which requires precise flexion of the neck while lying supine [13,14]. However, despite the high prevalence of neck pain and the knowledge of some potential mechanisms behind it, no consistently successful solution has been generated [15].

Targeted exercise is commonly prescribed as part of treatment for neck pain. The goal of exercise is often to strengthen the deep muscles associated with supporting the neck so that the surface muscles do not have to work as hard. Reduction in neck pain following exercise is sometimes accompanied by decreased activation of the superficial neck flexors during the CCFT [16]. In a recent meta-analysis of the effects of therapeutic exercise on neck pain and disability, a medium-sized effect on neck pain was found immediately after treatment and for up to 6 months after treatment. However, there was no effect of exercise on disability [17]. This suggests that for some people with neck pain, interventions that target motor behavior in daily life may be necessary to decrease pain-related disability. Furthermore, some people in pain may find exercise aversive, which may lead to low adherence [18]. Therefore, the discovery of an effective non-exercise program has the potential to benefit individuals experiencing neck pain and disability, including those who are either unwilling or unable to participate in an exercise regimen.

Alexander technique is a non-exercise approach aimed at improving modulation of postural muscle activity. Alexander students practice noticing and preventing excessive compression and straining, not just in the neck, but throughout the body [19,20]. It is thought that studying Alexander technique engages executive processes and spatial attention, leading to widespread changes in the adaptivity and distribution of postural tone throughout the musculoskeletal system [21].

Results from a large randomized controlled trial (RCT) indicate that a course of 24 one-to-one Alexander lessons is superior to both exercise and massage for low-back pain [22]. In a more recent RCT, 20 Alexander lessons in conjunction with usual care reduced neck pain compared to usual care alone [23]. In both aforementioned studies, benefits were retained or even enhanced 6 months to a year after lessons ended. Thus, individual Alexander lessons seem to be beneficial for people with chronic neck or back pain.

Although group Alexander classes are more affordable than private lessons, very few studies have examined their effectiveness [24]. A preliminary, uncontrolled study in our lab showed that group Alexander classes are a feasible and promising self-management approach for reducing neck pain [25]. The present study built on those data by assigning participants with neck pain to Alexander classes or exercise classes, to compare the effects of each intervention on neck pain, pain self-efficacy, posture, and neck muscle activity. This is the first study to compare Alexander group classes for neck pain to an active control group.

If Alexander classes facilitate reorganization of muscular control by reducing activation of superficial neck flexors [19,26], we predicted that participants in the Alexander group would show reduced activity of the superficial neck flexors during the CCFT and reduced neck pain after the intervention. If targeted exercise facilitates reorganization of neuromuscular control through strengthening of the deep cervical neck flexors leading to reduced load on the superficial neck flexors [16], then participants in the exercise group should also show reduced surface neck muscle activity during the CCFT and reduced neck pain after the intervention. If either intervention works through correcting postural alignment, that intervention should affect head–torso angle. If neck pain is associated with self-efficacy, neck angle, or activation of superficial neck flexors, we should see correlations between those metrics and neck pain.

## 2. Materials and Methods

### 2.1. Design

This study used a two-group, quasi-randomized, pre-test–post-test design with an exercise group and an Alexander group. Between the baseline data collection (pre-test) and the first post-intervention data collection (post-test), participants attended 10 exercise classes or 10 Alexander classes. There were 6 weeks between the pre-test and the first post-test, and 6 weeks between the first and second post-test. All experimenters were blinded to group assignment.

### 2.2. Participants

Participants were recruited via flyers, radio ads, and the University of Idaho employee newsletter. Sample size was determined by budget. Prospective volunteers were screened with an online survey; they were invited to participate if they scored 16% or higher on the Neck Disability Index [27], had at least 3 months of neck pain, were not currently receiving specialized care, and were available for the scheduled class times. Half of the participants were assigned based on which class time they could attend (Tues/Thurs or Mon/Wed) and the other half were assigned to balance the gender and neck disability between the groups. Participants did not know which class would be on which days of the week until after they were allocated to groups. Sixteen participants (9 women, 7 men, age 51 ± 17 years, mean 6 h/day sitting) completed all testing sessions and the intervention.

### 2.3. Interventions

Alexander technique classes were held from 6 to 7 p.m. on Tuesdays and Thursdays in a large music rehearsal room on the University of Idaho campus. The classes were designed and delivered by co-author SLC. Participants were taught principles and methods that would allow them to attend to unproductive habits of muscle tension and to become aware of their ability to make different choices. The classes included information on basic biomechanical and ergonomic principles (including anatomy of the neck, spine, and major joints of the upper and lower limbs) and the benefits of widening awareness to include both the self and the environment during activity. In addition, participants were guided in self-observation during common daily activities such as standing, sitting, computer work, personal care tasks, and household chores. Manual guidance was occasionally used, to demonstrate how to maintain a cohesive connection across regions of the body during activities [28]. On average, each participant received about 1 min of manual guidance per week. The Alexander teacher might place a hand (with permission) lightly on the participant’s head, neck, or back to help them become aware of some aspect of their body mechanics or to notice and release excess tension. Group activities such as tossing and catching were included to create an enjoyable structure for learning in a low-stakes context [29]. Each class began with 10 min for participants to share observations and ask questions. This was followed by 20 min of introduction to new material, 20 min of individual and group activities, and 10 min of discussion, questions, and planning of an individualized application of the principles and methods taught in that day’s class.

Exercise classes were held from 6 to 7 p.m. on Mondays and Wednesdays at a local fitness center. The exercises were designed by co-author TLM and administered by a trained instructor with experience working as a physical therapy technician. Participants performed exercises aimed at retraining use of the deep cervical flexors, strengthening postural muscles, and increasing range of motion. The retraining component was based on an adaptation of the protocol described by Jull et al. [13]. Participants placed the backs of their necks on a rolled towel and gently rotated their heads as if nodding ‘yes’, with a goal of activating the deep cervical flexors rather than surface neck flexors. Postural strengthening exercises included the use of dumbbells and resistance bands (Theraband^®^, Akron, OH, USA) to target the trapezius and upper-back muscles. The trapezius and anterior neck muscles were stretched to increase mobility. Each exercise class included 10 min of light stretching, followed by 45 min of retraining and strength exercises aimed at promoting a more upright posture. Verbal instructions and movement demonstrations were given by the instructor for the first session and were repeated as needed in subsequent sessions. Participants were encouraged to minimize contraction of superficial neck and trapezius muscles while performing the shoulder and upper-back strengthening exercises. Manual guidance was only used if the participant was unable to perform the exercise as instructed. At the beginning of the exercise intervention, subjects picked their own dumbbell weights and resistance bands based on their ability to complete the exercises as instructed. Supine horizontal abduction and scapular retraction were performed using resistance bands, and shoulder and upper-back exercises were performed with dumbbells. Correct exercise execution was monitored and occasionally cued (verbal and manual) by the instructor, and participants were encouraged to increase the resistance so long as they were not compensating by recruiting unwanted muscle groups (sternocleidomastoids, trapezius).

Both instructors offered a single make-up class for students who had to miss a regular class session.

### 2.4. Outcomes

#### 2.4.1. Protocol

All methods were carried out in accordance with relevant guidelines and regulations. Testing took place in the Mind in Movement Laboratory on the University of Idaho campus. Upon arrival to the lab for the first visit, participants consented to take part in this study according to a protocol approved by the University of Idaho’s institutional Review Board. The rest of the visit was the same each time. Participants completed self-report measures, then performed the CCFT with surface electromyography, then played a video game while their posture was video recorded. At completion of the second post-test, participants were paid $50.

#### 2.4.2. Self-Report Measures

Our primary outcome measure was the Northwick Park Questionnaire (NPQ), a 9-item questionnaire assessing severity of neck pain and disability during everyday activities [30]. Each item is scored from 0 to 4 and then summed, for a total score between 0 and 36 with higher scores indicating worse pain. This was converted to a percentage score to allow comparisons with other studies.

We also administered the Pain Self-Efficacy Questionnaire (PSEQ), a 10-item questionnaire assessing confidence regarding performance of daily activities despite neck pain [31]. Each item is scored using a 7-point Likert scale, where 0 = not confident at all and 6 = completely confident; item scores are summed, for a total score between 0 and 60 with higher scores indicating greater confidence.

At the second post-intervention assessment, we administered a survey about participants’ experience of the class series they participated in and its effect on their life.

##### CCFT with Electromyography.

Prior to electrode placement, skin was prepped by shaving any hair, lightly abrading with sandpaper tape (3M^®^, St. Paul, MN, USA), and cleansing with 70% isopropyl alcohol. Single Bagnoli^®^ DE-2.1 Ag-AgCl electrodes (Delsys^®^, Natick, MA, USA) were placed bilaterally on the sternocleidomastoids approximately 2/3 of the way down the muscle, close to the manubrium. Electrode placements were obtained from previous studies of the CCFT [14] and were based on palpation during supine neck raises and rotations. Data were collected and pre-processed with *The MotionMonitor*^®^ Classic software (Innovative Sports, Chicago).

Following electrode placement, participants performed a reference voluntary contraction while lying supine. Electrode placement was adjusted if the power spectral density of the signal (PSD) showed a peak at 60 Hz with limited power content from other frequencies. The reference value was obtained by having participants hold their heads approximately 3 inches off the floor for 10 s while muscle activity was recorded. Three reference contractions were recorded to ensure a reliable measurement and later averaged during data analysis.

The CCFT was administered using a standard clinical protocol described by Jull et al. [13]. Participants lay supine, with a pressure biofeedback unit (Chattanooga^®^, Chattanooga, TN, USA) under the neck touching the external occipital protuberance to provide visual feedback to the participant and experimenter. The pressure sensor responded to the slight retraction of the neck normally caused by contraction of the deep cervical flexors [32]. The sensor was inflated to 20 mmHg at baseline, and participants practiced obtaining the 5 required pressure levels in 2 mmHg increments (22–30 mmHg). The CCFT consists of all 5 levels in ascending sequence, with participants instructed to hold each level for 10 s while muscle activity is recorded. Electromyography recording was initiated when the participant reached a stable and accurate pressure. Three trials were recorded at each level, and participants rested for 30 s between each trial. After each trial, pressure was returned to 20 mm.

##### Video Game with Posture Photos.

**Figure 1.**
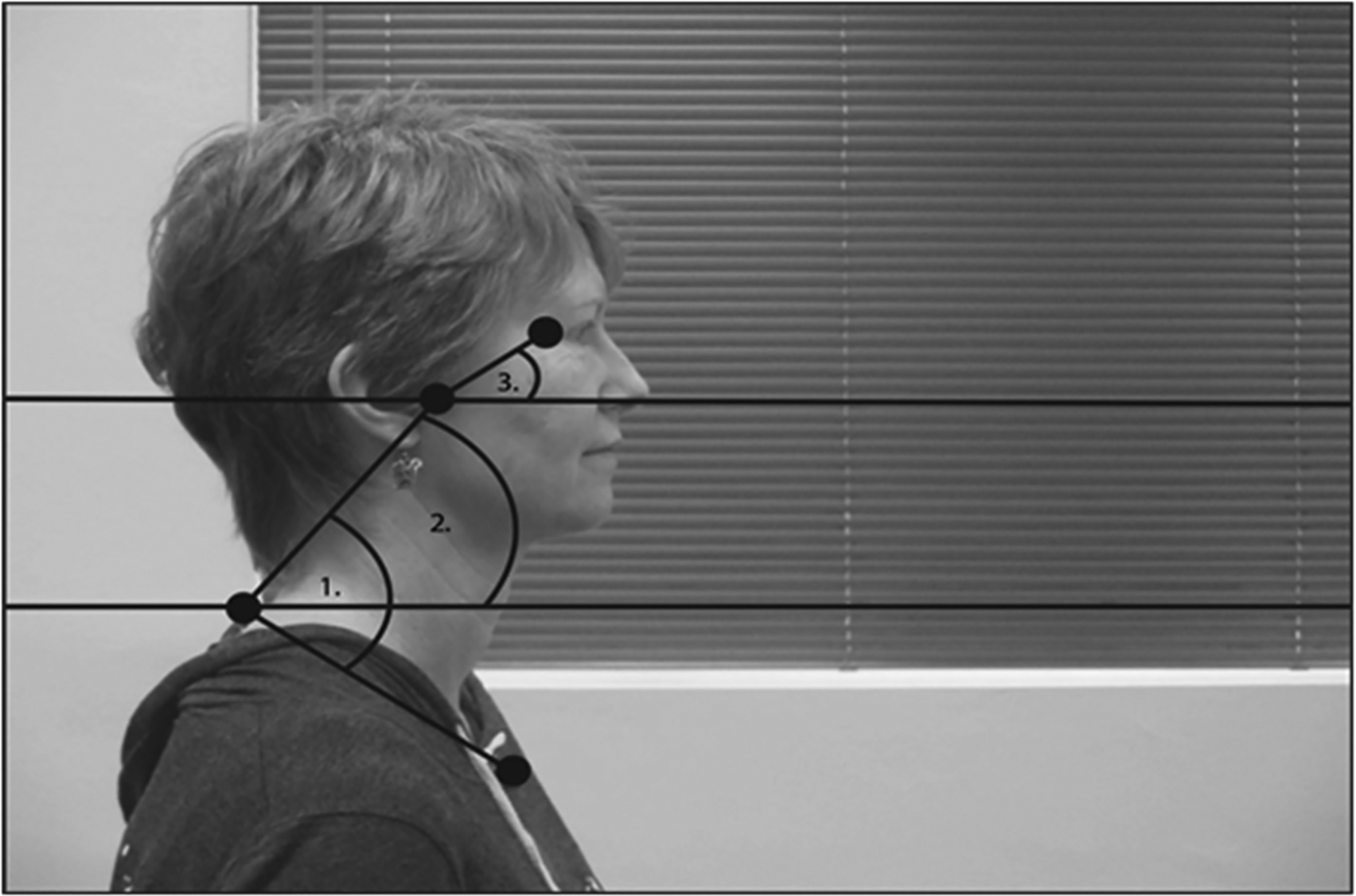
Diagram of angles used for posture analysis. (**1**) Head–torso angle was defined based on the angle between tragus, C7, and manubrium. (**2**) Neck angle was defined based on the angle between tragus, C7 and horizontal plane. (**3**) Head angle was defined based on the angle between the orbit, tragus and horizontal plane.

While seated, participants played Diner Dash^®^ (Play-first, San Francisco, CA, USA), a computer game with simple rules, for 5 min. The game involves pointing and clicking with a mouse to serve patrons in a virtual restaurant. Difficulty increases with continued play, as more virtual patrons arrive. Participants read the instructions and practiced for 5 min before recording commenced. Reflective markers were placed at specific landmarks (tragus, spine of 7th cervical vertebra, and manubrium) used to measure posture—see Figure 1. Two-dimensional static images were automatically recorded once per minute using a video camera on a tripod to allow us to examine position in the sagittal plane. Before participants began playing, the experimenter adjusted the chair, table, and monitor positions in accordance with the participant’s anthropometry in compliance with standards set by the U.S. Department of Labor, Occupational Safety and Health Administration [33].

### 2.5. Data Reduction

We analyzed postural alignment from still images using the ImageJ software (National Institutes of Health, Bethesda, MD, USA). Initially, we only intended to look at head–torso angle (shown as ‘1’ in Figure 1). However, those results were surprising, so we also analyzed neck and head angles with respect to horizontal (shown as ‘2’ and ‘3’ in Figure 1).

We analyzed electromyography data using a custom script written in MATLAB^®^ (Mathworks, Natick, MA, USA). Muscle activity amplitude was obtained by calculating the root mean squared value of each signal over 50 ms windows and then averaging across all windows. Amplitude was averaged across the 3 trials at each level of the CCFT and expressed as a percentage of reference values. Amplitudes for left and right sternocleidomastoid were compared. Since no difference was found, left and right values were averaged. We also computed the median frequency of the power spectral density of the muscle signal at each level of the CCFT, as this has been shown to decrease commensurately with fatigue [34].

### 2.6. Statistical Analysis

Statistics were analyzed in Prism (GraphPad Software, San Diego, CA, USA), with type I error set at 0.05. We examined baseline characteristics of the 2 groups with t-tests and chi-square tests to ensure equivalency. Before each parametric test, the Shapiro–Wilk test for normality was run to determine whether the distribution of the data was normal. All the sets of data passed this test. A 2-way repeated-measures analysis of variance (ANOVA) was used to analyze each outcome measure: self-report, posture, EMG amplitude, and EMG frequency. The factors were group and session. For the EMG analyses, we ran separate ANOVAs at Level 1 and Level 5. To further evaluate the interactions of interest, we made pairwise comparisons and controlled for multiple comparisons with Tukey’s post hoc adjustment. To assess possible mechanisms responsible for improvement, we examined relations between changes in neck pain before and after the intervention and changes in other outcome measures, using Pearson’s product–moment correlation. We also tested for a correlation between posture and muscle activation.

## 3. Results

### 3.1. Recruitment, Screening, and Attendance

**Figure 2.**
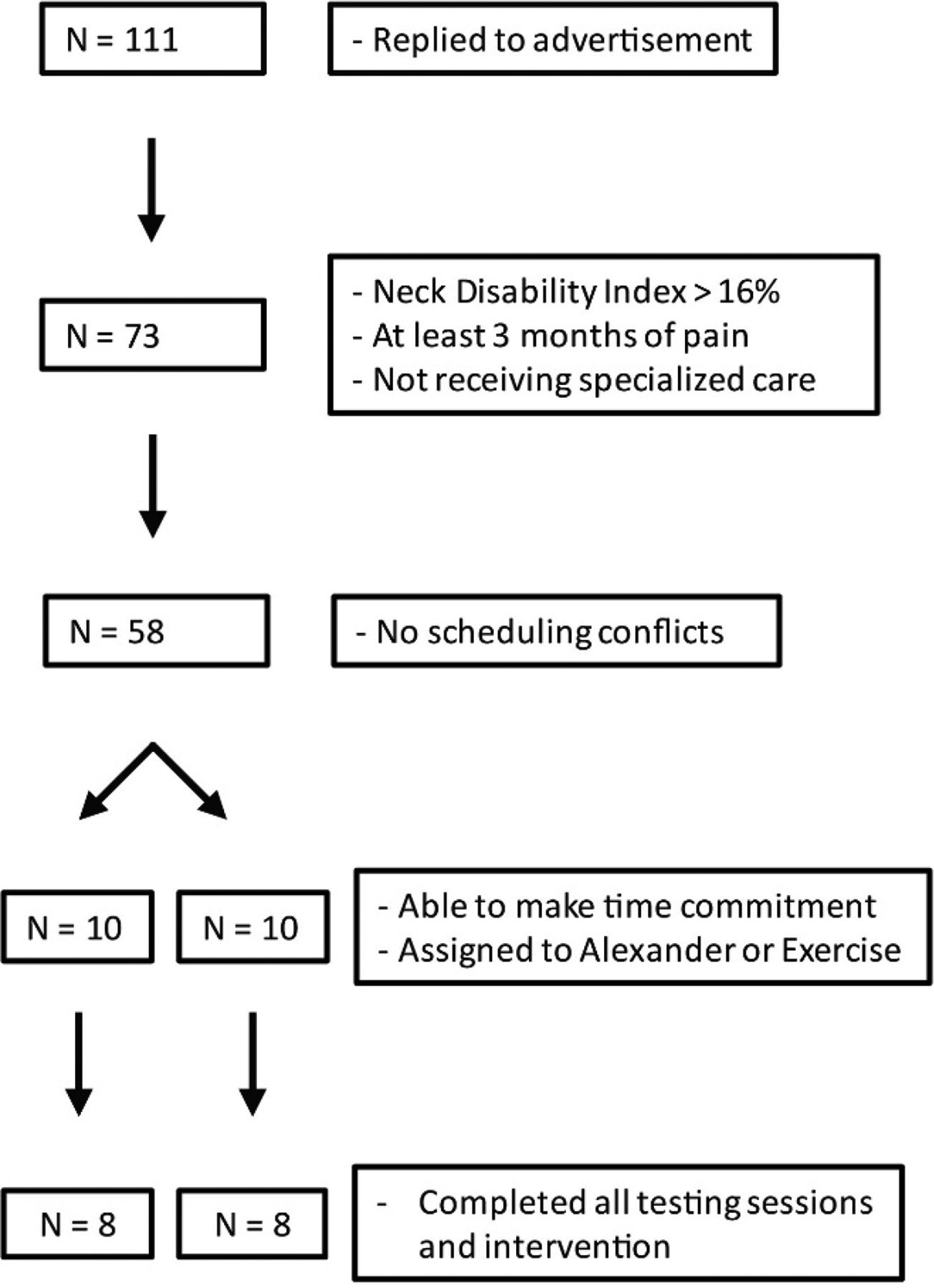
Flow chart of participant recruitment and attrition.

Figure 2 shows the flow of participants through the recruitment and retention process. One hundred eleven people responded to our flyer and filled out our online survey. Thirty-eight were excluded for insufficient pain or because they were currently receiving specialized treatment. Fifteen were excluded based on scheduling conflicts, and 38 were unable to make the time commitment required. Twenty participants were assigned to Alexander technique or targeted exercise classes (pseudorandom assignment to accommodate scheduling needs; participants were not informed of which course would be offered on which days).

In the Alexander group, two participants declined to attend any of the intervention sessions due to last-minute scheduling concerns. In the exercise group, one person dropped out after the first session because of pain and one person declined to attend any of the intervention sessions. All 16 participants who participated in the interventions completed all 3 testing sessions. Table 1 shows the baseline demographic information for each participant who completed all testing sessions and the intervention.

### 3.2. Self-Reports: Neck Pain, Pain Self-Efficacy, and Course Surveys

**Figure 3.**
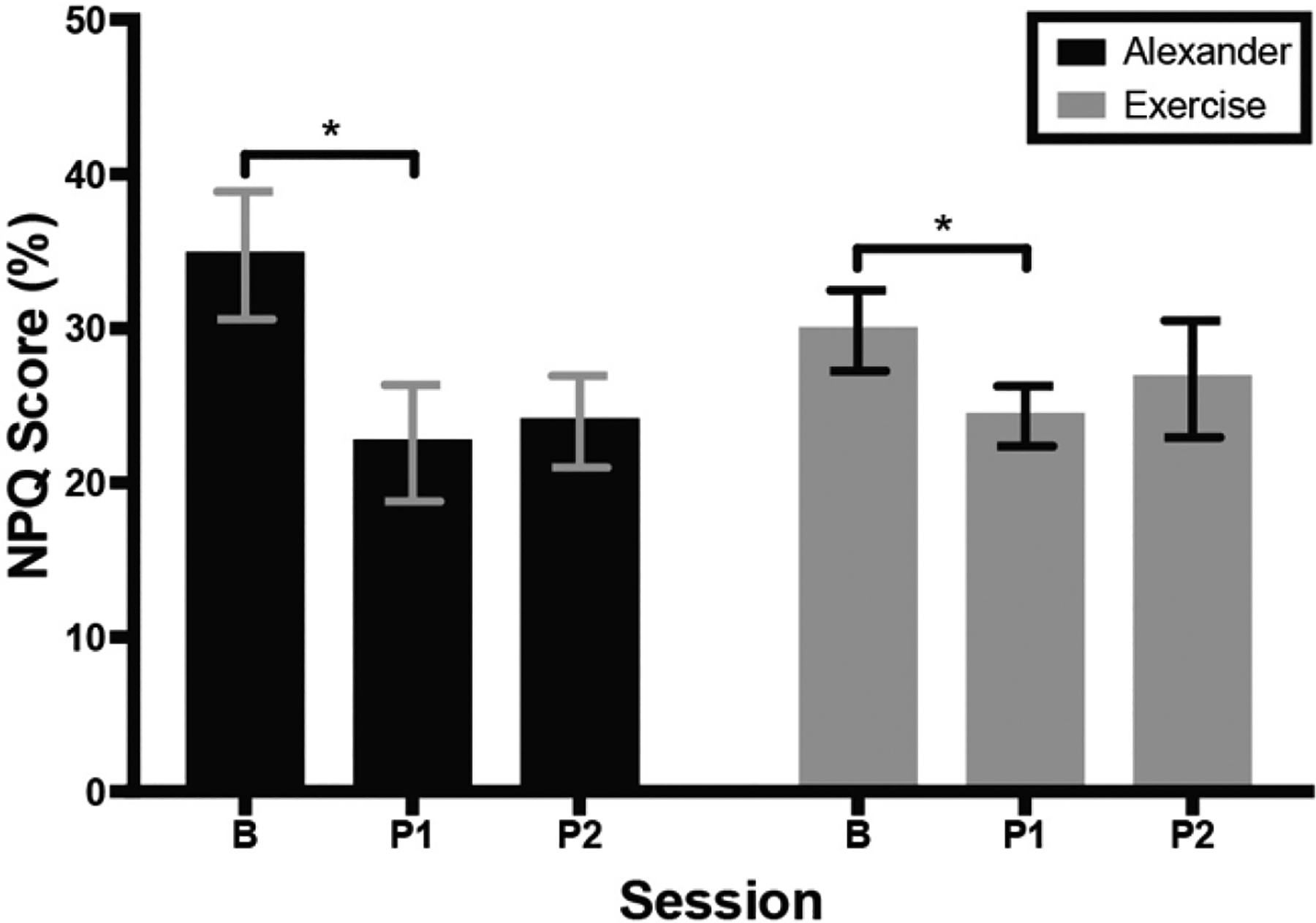
Score on Northwick Park Questionnaire (NPQ) across groups and testing sessions. Higher score; indicstes more neck pain and associated disability. Error bars are the standard error of the mean. B = baseline; P1 = 1st post-test; P2 = 2nd post-test. * indicates significant difference (*p* < 0.05) between baseline and first post-test.

Figure 3 shows average NPQ scores of the Alexander and exercise groups across testing sessions. Eleven out of 16 participants reported a decrease in pain/disability immediately after the intervention, with a significant effect of session; F(1.6, 23.5) = 5.9, *p* = 0.01, eta^2^ = 0.16, Greenhouse–Geisser (ε = 0.84). There was no difference between groups. Post hoc comparisons showed that NPQ score was smaller at the first post-test than at baseline, *p* = 0.02. In the Alexander group, 6 out 8 participants reported a decrease in pain/disability immediately after the intervention, with an overall decrease of 11% and a 31% reduction from baseline. This improvement exceeds the threshold for minimum clinically important difference [35]. In the exercise group, 5 out of 8 participants reported a decrease in pain/disability immediately after the intervention, with an overall decrease of 5% and a 16% reduction from baseline. (This does not exceed the threshold for clinical significance [35].) No interactions were significant.

**Figure 4.**
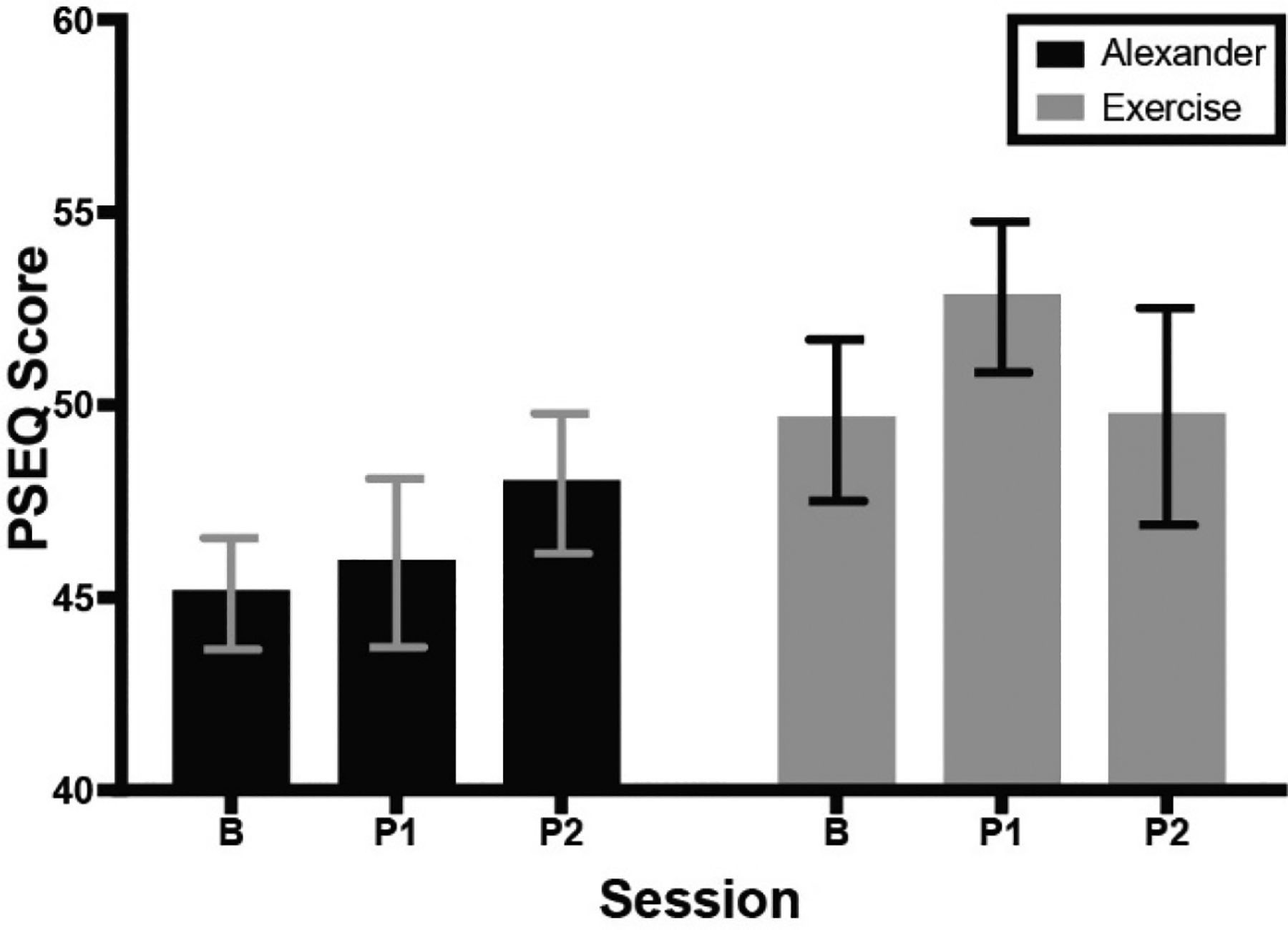
Score on Pain Self-Efficacy Questionnaire (PSEQ) across groups and testing sessions. Higher score indicates higher self-efficacy. Errors bars are the standard error of the mean. B = baseline; P1 = 1st post-test; P2 = 2nd post-test.

Figure 4 shows average PSEQ scores of the Alexander and exercise groups across testing sessions. PSEQ score was not significantly different across testing sessions or between groups, nor was there an interaction.

Tables 2 and 3 show the results of the post-intervention course surveys. Participants in both groups indicated that they enjoyed the classes and that they enjoyed the social aspect of the classes. Compared to participants in the exercise group, participants in the Alexander group agreed more strongly that they: learned how their movement contributed to their neck pain, t(15) = 2.4, *p* = 0.03; were surprised by the things they learned, t(15) = 3.0, *p* = 0.008; applied what they learned in class to an activity practiced in class, t(15) = 3.5, *p* = 0.003, and noticed themselves using their usual way of doing something and choosing a different way, t(15) = 3.0, *p* = 0.009.

### 3.3. Posture

**Figure 5.**
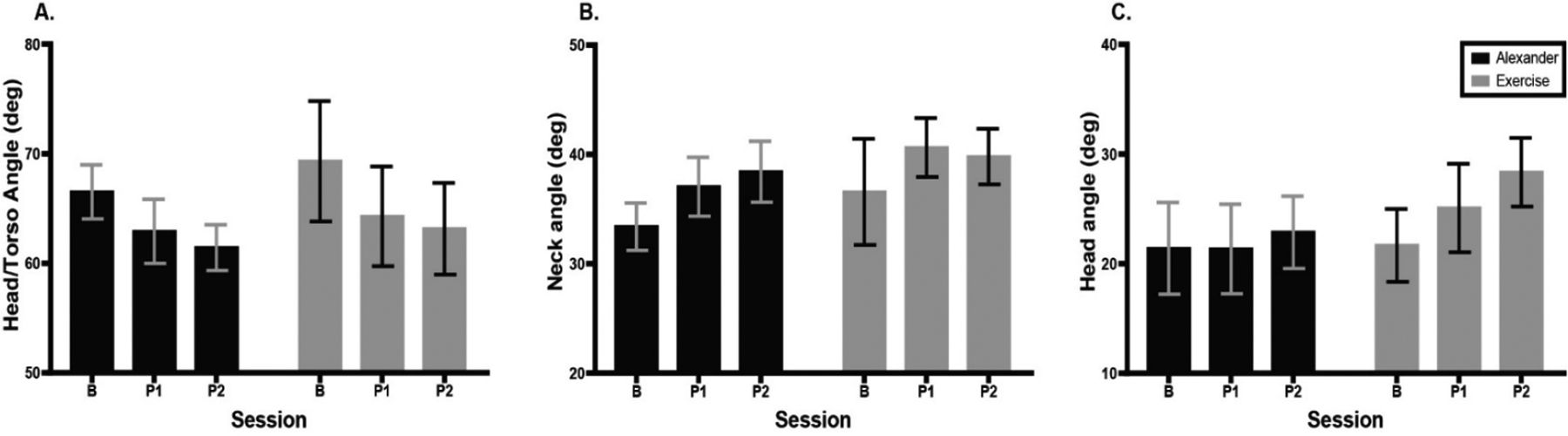
Comparison of posture during video-game task computed during 5 min play across groups and testing sessions. Errors bars are the standard error of the mean. (**A**) Head–torso angle (tragus, C7, manubrium). Higher angle indicates less forward head posture. (**B**) Neck angle (tragus, C7, horizontal). Higher angle indicates head farther back in space. (**C**) Head angle (orbit, tragus, horizontal). Higher angle indicates head extension. Error bars are the standard error of the mean. B = baseline; P1 = 1st post-test; P2 = 2nd post-test.

Figure 5 shows average head–torso angle, neck angle, and head angle during the 5 min computer task for the Alexander and exercise groups across testing sessions. Head–torso angle (Figure 5A) revealed a main effect of session; F(1.48, 20.78) = 5.0, *p* = 0.02, eta^2^ = 0.03, Greenhouse–Geisser (ε = 0.74). Post hoc tests indicated that head–torso angle was smaller at the second post-test than at baseline, *p* = 0.002. Neck angle (Figure 5B) revealed a main effect of session; F(1.42, 19.8) = 5.8, *p* = 0.02, eta^2^ = 0.05, Greenhouse–Geisser (ε = 0.71). Post hoc tests indicated that neck angle in the Alexander group was larger at the second post-test than at baseline, p = 0.03. Head angle (Figure 5C) revealed a main effect of session; F(1.5, 21.1) = 4.2, *p* = 0.04, eta^2^ = 0.03, Greenhouse–Geisser (ε = 0.75). Post hoc tests indicated that the head angle was larger at the second post-test than at baseline, *p* = 0.007.

### 3.4. Sternocleidomastoid Activation

One participant’s electromyography data were not analyzed due to difficulty acquiring a clean signal.

**Figure 6.**
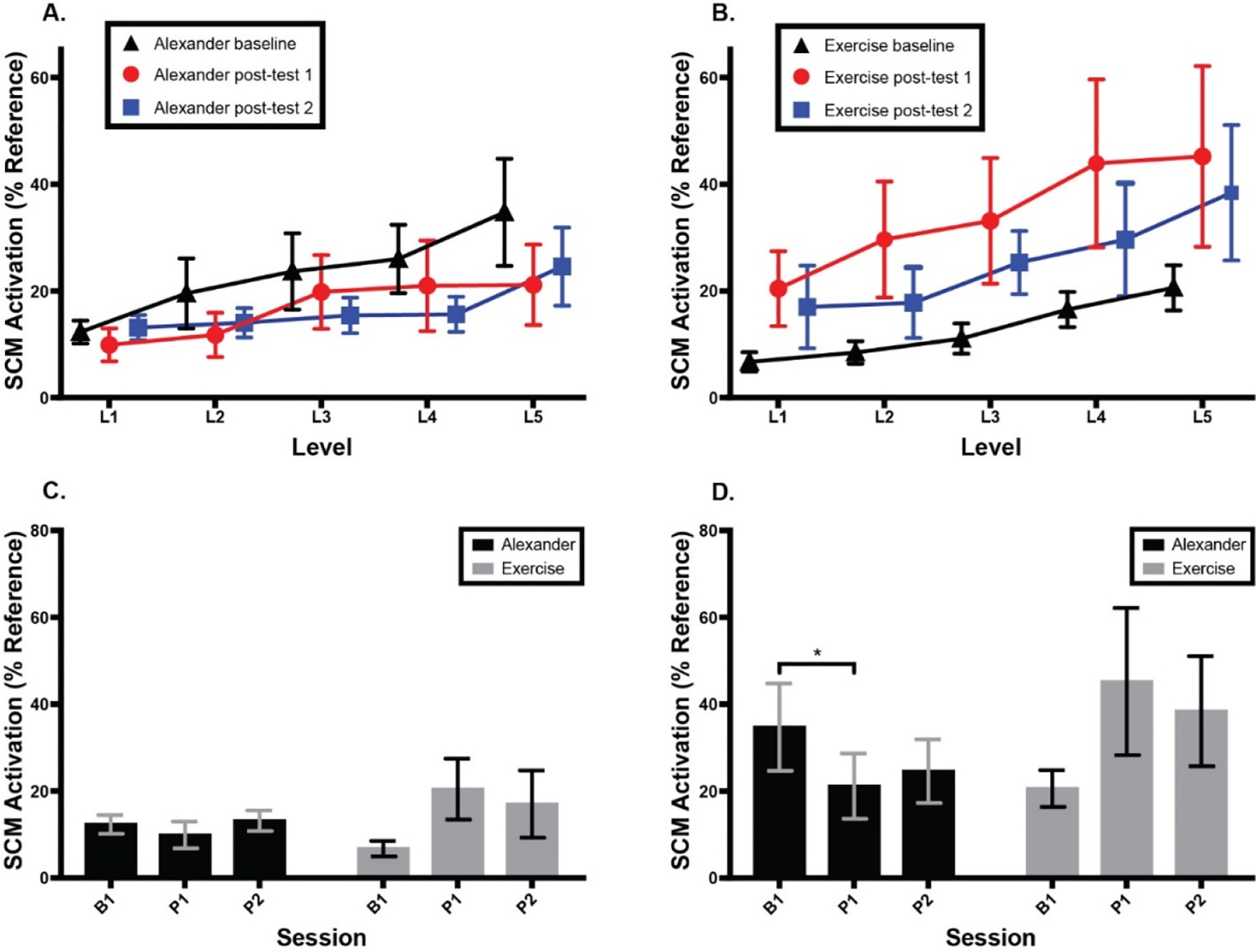
Normalized bilateral sternocleidomastoid (SCM) muscle activity as a percent of accepted voluntary contraction during cranio-cervical flexion test (CFFT). (**A**) Alexander group SCM activity across three testing sessions and five CCFT levels. (**B**) Exercise group SCM activity across three testing sessions and five CCFT levels. (**C**) SCM activity across groups and sessions at CCFT Level 1. (**D**) SCM activity across groups and sessions at CCFT Level 5. Error bars indicate the standard error of the mean. * signifies *p* < 0.05. B1 = baseline; P1 = 1st post-test; P2 = 2nd post-test.

Figure 6 shows SCM activation as a percentage of reference voluntary contraction for the Alexander and exercise groups across testing sessions and CCFT levels. There was no main effect or interaction at Level 1 of the CCFT. At Level 5, there was no main effect of session or group, but there was a significant interaction between session and group, F (2, 26) = 6.6, *p* = 0.005, eta^2^ = 0.08, with the Alexander group showing a significant decrease in SCM activation following the intervention (Tukey post hoc, *p* = 0.047) and the exercise group showing a non-significant *increase* in SCM activation.

**Figure 7.**
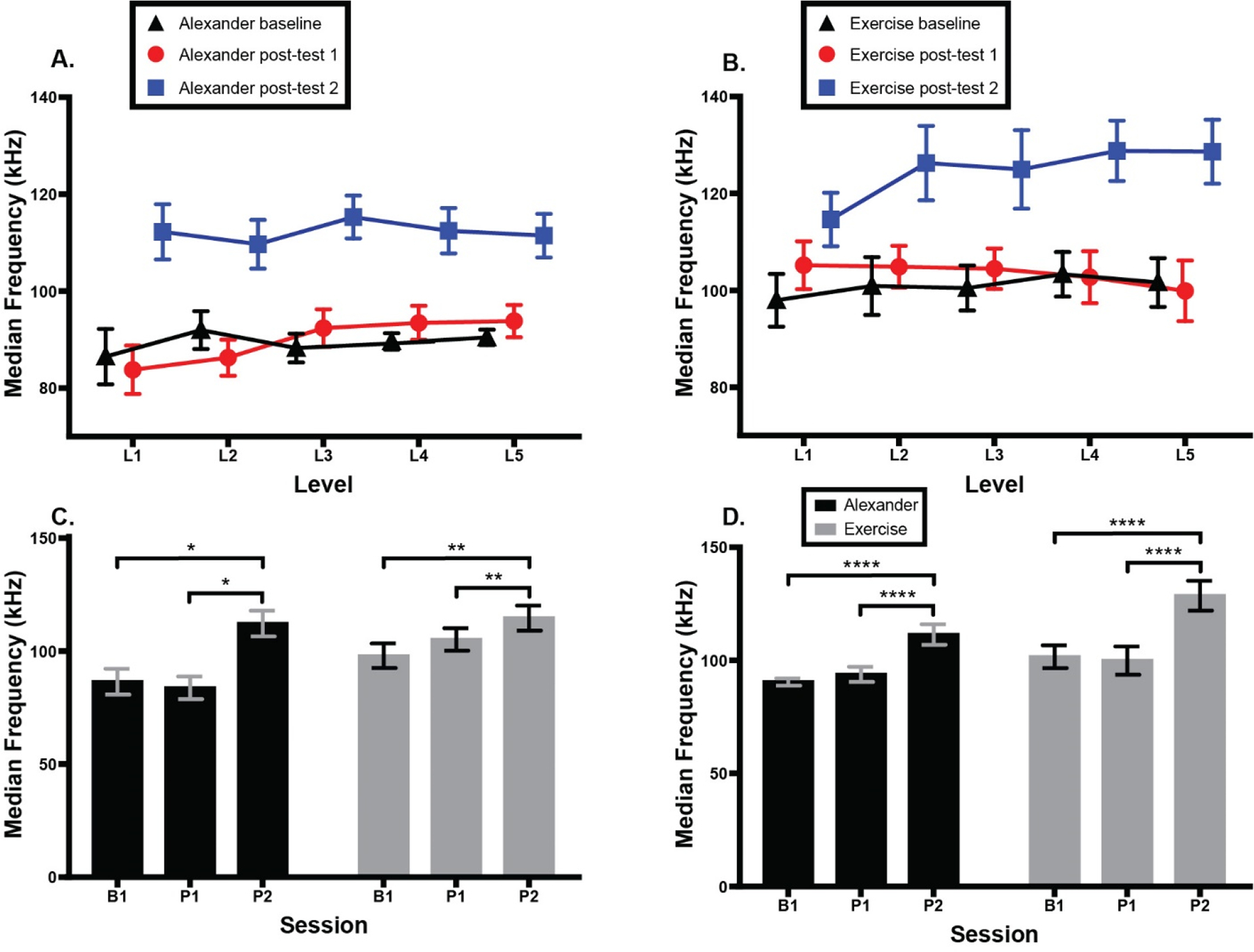
Median frequency of bilateral sternocleidomastoid (SCM) muscle activity during cranio-cervical flexion test (CFFT).). (**A**) Alexander group SCM activity across two groups, three testing sessions, and five CCFT levels. (**B**) Exercise group SCM activity across groups, sessions, and CCFT levels. (**C**) SCM activity across groups and sessions at CCFT Level 1. (**D**) SCM activity across groups and sessions at CCFT Level 5. Error bars indicate the standard error of the mean. Higher SCM activation indicates more muscle activity. * signifies *p* = 0.01. ** signifies *p* = 0.007. **** signifies *p* < 0.0001. B1 = baseline; P1 = 1st post-test; P2 = 2nd post-test.

Figure 7 shows median frequency of SCM muscle activation across groups, testing sessions, and CCFT levels. At CCFT Level 1, there was a main effect of session; F(1.65, 23.02) = 9.0, *p* = 0.002, eta^2^ = 0.26, Greenhouse–Geisser (ε = 0.82), and a main effect of group; F(1, 14) = 7.6, *p* = 0.02, eta^2^ = 0.10, but no interaction. Post hoc comparisons revealed a higher frequency at second post-test than either baseline (*p* = 0.02) or first post-test (*p* = 0.007). Overall, frequencies were higher in the exercise group than in the Alexander group. At CCFT Level 5, there was a main effect of session; F(1.91, 26.74) = 34.9, *p* < 0.0001, eta^2^ = 0.38, Greenhouse–Geisser (ε = 0.96), but no group difference or interaction. Post hoc tests revealed a higher median frequency at the second post-test than at both baseline, *p* < 0.0001, and first post-test, *p* < 0.0001.

### 3.5. Correlations

**Figure 8.**
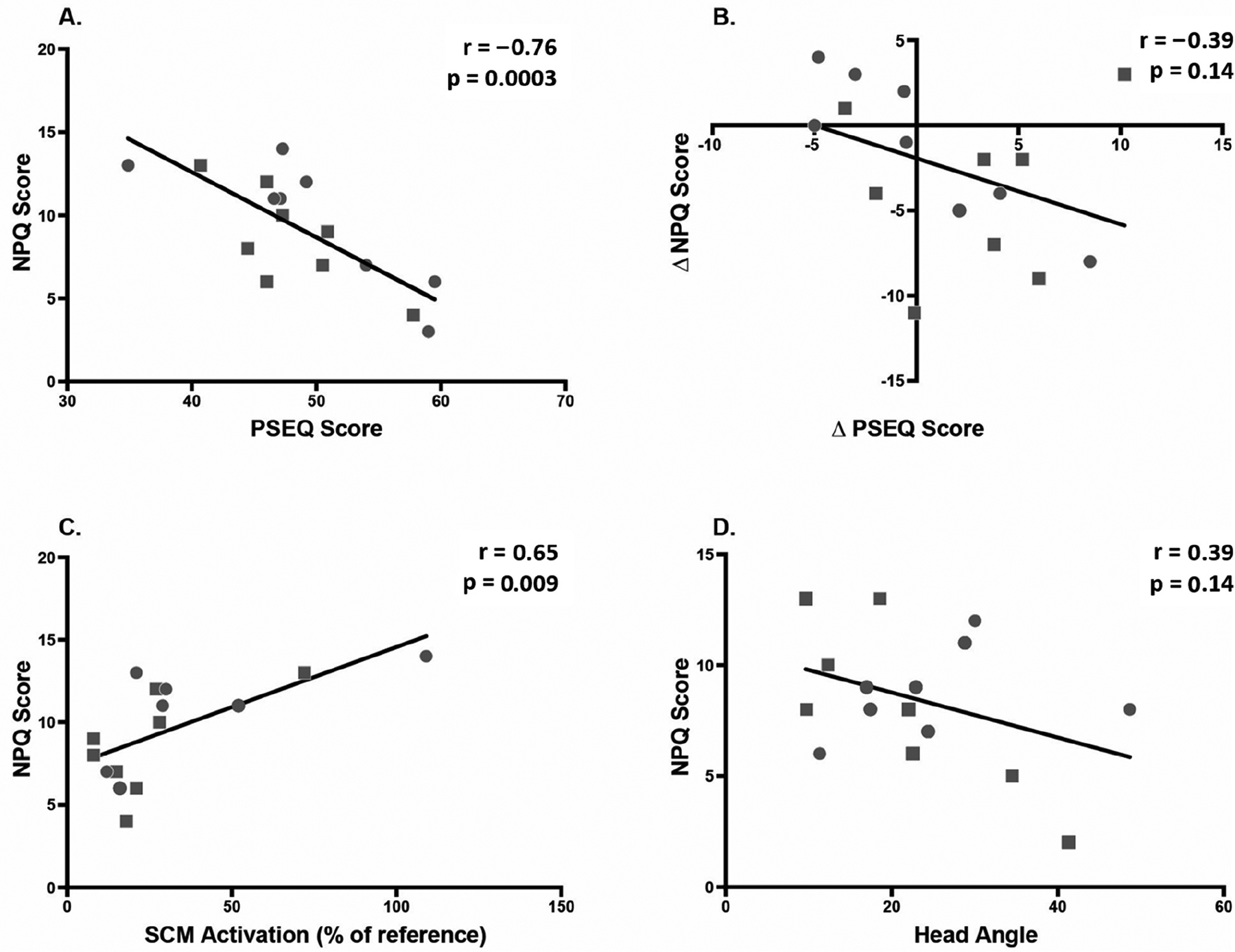
Correlations between predictor variables and neck pain (NPQ score). Squares indicate Alexander group; circles indicate exercise group. (**A**) Correlation between pain self-efficacy (PSEQ) and neck pain ait 2nd post-test, indicating tin at higher self-efficacy is associated with lower pain. (**B**) Correlation between change in pain self-efficacy (PSEQ) and change in neck pain between Baseline and 2nd post-test, indicating that a decrease in neck pain is associated with an increase in self-efficacy. (**C**) Correlatinn between sternocleidomastoid (SCM) activation and neck pain at 2nd post-test. (**D**) Correlation between head angle and neck pain (NPQ) at 1st post-test.

Table 4 shows correlations of NPQ score and change in NPQ score with other outcome measures. Figure 8 shows scatter plots of the relationships with the highest correlations. At second post-test, NPQ score correlated significantly with PSEQ score (r = −0.76) and neck muscle amplitude (r = 0.65). There were two non-significant correlations of note: change in NPQ with change in PSEQ (r = −0.39) and NPQ with head angle at P1 (r = −0.39). There was no correlation between posture and muscle activation.

Results are presented as r(p). NPQ = Northwick Park Questionnaire (neck pain). PSEQ = pain self-efficacy questionnaire. Head–torso angle = [tragus, C7, manubrium] angle during computer game. Neck angle = [tragus, C7, horizontal] angle during computer game. Head angle = [orbit, tragus, horizontal] angle during computer game. Muscle activity: amplitude = amplitude of electromyography signal from bilateral sternocleidomastoid, normalized to reference contraction. Muscle activity: frequency = median frequency of electromyography signal from bilateral sternocleidomastoid at CCFT Level 1. Scatter plots for bolded correlations are shown in Figure 8.

## 4. Discussion

### 4.1. Summary of Results

This study used a two-group pre-test–post-test design to compare the effects of two different interventions (Alexander technique and targeted exercise) on neck pain, pain self-efficacy, postural alignment, and neck muscle activity. The results indicated that 10 Alexander classes were at least as effective as 10 exercise classes at attenuating neck pain. Both groups reported significantly decreased neck pain immediately after the intervention, with no significant increase in pain 5 weeks after the intervention ended.

Self-efficacy has been considered a plausible mediator of neck pain [36]. We found no effect of either intervention on self-efficacy. However, this could have been a type II error due to small sample size. We saw no relation between self-efficacy and neck pain at baseline, but 5 weeks after the intervention ended, self-efficacy was strongly correlated with neck pain, and the relationship between increased self-efficacy and decreased neck pain after the intervention approached significance. There was no difference in self-efficacy between the groups, but participants in the Alexander group were more likely than those in the exercise group to report that they learned how their movement habits contributed to their neck pain and that they were able to choose different ways to carry themselves. This suggests that Alexander technique teaches generalizable skills that can be applied in everyday motor tasks and thus may be a viable long-term approach to neck pain.

Contrary to our prediction, head–torso angle during the video game decreased after the intervention in both groups. We therefore also examined neck and head angles separately with respect to the horizontal plane. The fact that neck angle *increased* indicates that the decreased head–torso angle was not due to a more forward neck. Instead, it appears that the upper torso became more vertical after the interventions. Unfortunately, we did not record landmarks that allow us to confirm this conclusion. Unofficial post hoc tests (not driven by a significant interaction) comparing baseline to second post-test in each group suggest that the increase in neck uprightness was driven by the Alexander group (*p* = 0.03), while the increase in head extension was driven by the exercise group (*p* < 0.0001). This provides a clue that the two interventions may have quite different mechanisms. However, none of the posture angles were significantly correlated with neck pain or change in pain. This absence of relationship is consistent with our previous work [25], and with a growing body of literature suggesting that the alignment aspect of posture may not be the most important determinant of pain [37].

After the intervention, the Alexander group showed significantly decreased activity in surface neck muscles during the neck flexion task, while the exercise group showed a tendency toward *increased* activity in the same muscles. These changes in SCM activation were still present 5 weeks after the intervention. In addition, 5 weeks after the intervention ended, lower SCM activation was associated with lower neck pain. This result is in line with the emphasis in Alexander technique on learning to observe and inhibit habitual patterns of reaction in daily life [28,32,38].

Both groups showed an increase in muscle firing frequency after the intervention, thought to reflect decreased muscle fatigue. The effect was strongest at the last testing session and at the most difficult level of the neck flexion task. However, EMG frequency did not correlate with neck pain.

### 4.2. Relation to Prior Work

Previous studies have shown that one-to-one Alexander lessons can lead to reductions in knee pain [19], back pain [22], and neck pain [23]. In particular, the ATLAS study, a large RCT of Alexander technique for chronic neck pain, found that a series of twenty 30 min lessons reduced NPQ score by about 11%—the same reduction in neck pain/disability that we found after this Alexander technique group class [23]. The ATLAS study found an additional decrease in pain 6 months later. The absence of further decline in pain after the course ended in the present study suggests that 10 classes may be enough to immediately decrease neck pain but not enough to instill long-term habits that will promote further improvement. Interestingly, the 4% reduction in NPQ reported for the control group in the ATLAS study is comparable to the result seen here in our exercise group and to the 5% NPQ reduction seen in other brief exercise studies [39].

The retention rate for our group Alexander class was also comparable to that seen in one-to-one lessons, with a few people dropping out at the beginning and a very high attendance rate for the remaining students [25]. We also found ratings of class enjoyment similar to those seen in other populations [40,41]. Converging evidence suggests that group Alexander classes are a highly acceptable intervention.

The ATLAS study, unlike the present study, found a substantial increase in pain self-efficacy following Alexander lessons [39]. This could be due to participant differences (to qualify for the ATLAS study, participants had to score 28% on the NPQ, whereas a score of 16% was sufficient to qualify for the present study), difference between group and one-to-one sessions, the longer duration of the ATLAS class series (20 weeks vs. 5 weeks in the present study), or simply their larger sample size. Considering our finding that participants in the Alexander group were more likely than those in the exercise group to report that they learned how their movement habits contributed to their neck pain and to choose different ways to carry themselves, it may be that it takes longer than 5 weeks for that learning to translate into an increase in pain self-efficacy. This explanation is supported by the numerical tendency for self-efficacy to increase between the first and second post-test in the Alexander group but not in the exercise group, and by the increasing correlation between NPQ and PSEQ across the three testing sessions.

Alexander classes led to reduced activation of SCM, a muscle whose overactivation has been implicated in neck pain [13]. This is consistent with our previous study of group Alexander work, which found a near-significant reduction in SCM activation. The contrasting increase in SCM activation in the exercise group was surprising. Previous work has found that some exercise programs focusing on training the motor control and coordination of cervical flexors resulted in a decrease in SCM activation during the CCFT, while others had no effect [16,42]. It is worth noting that exercise programs vary in their content and emphasis. For instance, the program of Jull et al. [16] focused on training the coordination and control of the cervical flexors using constant direct feedback by a physiotherapist, while our program included feedback to participants only during the brief retraining portion of the exercise class. Feedback was only given if the subject was clearly overusing the SCM muscles or performing the exercise incorrectly. This leads to an interesting question about the amount of manual feedback needed to see positive results in patients with neck pain with different interventions. Participants in both groups had minimal individualized attention from the instructor, but only the Alexander class led to a reduction in SCM activation, suggesting that the indirect, whole-body approach of the Alexander technique may be more effective than the direct, neck-focused approach of the targeted exercise, particularly in a group class context with limited manual contact. This conclusion is consistent with a large body of work demonstrating that instructions focused directly on a specific body part are less effective than indirect instructions [43,44].

In the present study, both interventions led to an increased frequency of surface neck muscle firing during the CCFT, consistent with a decrease in fatigue and with our previous findings [25]. Our previous study using the same Alexander class format found a correlation between increasing median frequency of SCM activity and decreasing pain. The absence of that correlation in the present results is puzzling. Again, we speculate that the discrepancy may result from participant differences, as inclusion for the prior study required a history of 6 months of pain (and thus may have been more truly “chronic”) while the present study only required 3 months. In support of this explanation, median frequencies were overall much lower (suggesting greater fatigue) in the previous study than in the present study.

### 4.3. Strengths, Limitations, and Future Directions

This was a two-group pre-post-post study. The inclusion of two active treatment groups was a strength of this study, as was the replication of the same class protocol previously tested. The small sample size was a weakness. In addition, the second post-test was only 5 weeks after the intervention ended, so long-term benefits are unclear.

In contrast with our previous study, this study included participants who had only been in pain for 3 months (rather than a minimum 6) and those who had recently ceased specialized treatment [27]. These changes were made to improve recruitment. However, they may have reduced effect sizes.

The Alexander intervention was delivered by a certified teacher, whereas the exercise intervention was delivered by a more junior professional. From one perspective, this could seem to bias this study in favor of the Alexander intervention. However, realistically, Alexander classes can only be delivered by trained Alexander teachers, while exercise classes are often delivered by people with less training.

The two classes met in different spaces. It is possible that one room had a more favorable impression than the other, but this is unlikely based on the equivalent ratings for the spaces. (See Table 2A, question 14.)

This 5 week, 10 lesson structure was chosen to balance the time-commitment of the participants with the desired strength of the manipulation. Other studies have shown that more sessions lead to a stronger result with better retention. The BMJ study of Alexander lessons for low-back pain found that those who had 6 lessons did only 41% as well as those who had 24 lessons [22].

Future studies should include larger samples, longer-duration chronic pain, longer interventions, and longer follow-up. In addition, the different EMG results between the two interventions suggest that the mechanisms of Alexander technique and targeted exercise may be distinct, which leads to the question of whether combining exercise and Alexander work would lead to even larger benefits for people with neck pain. Previous work has found that combining Alexander work with walking led to increased benefit for people with chronic low-back pain [22]. This suggests that the benefits of Alexander technique and exercise may be additive.

## 5. Conclusions

The findings of this study, while preliminary, support the growing body of evidence that Alexander technique is an effective and feasible non-exercise alternative to the treatment of neck pain. Because exercise is not realistic or appealing for everybody, it is important to show that non-exercise interventions can be successful. The findings reported here suggest that Alexander classes are at least as good as exercise classes for reducing neck pain and associated disability. Both classes were rated as enjoyable. The retention and pain reduction results from the Alexander group intervention compare well to benefits seen in a larger study of one-to-one Alexander lessons, suggesting that this more cost-effective delivery method is acceptable and effective. Surface neck muscle activation decreased in the Alexander group but not in the exercise group, suggesting that the two approaches work via different mechanisms. Larger studies are justified, especially to investigate the possible additive benefits of Alexander technique and exercise.

## Figures and Tables

**Table 1.** Demographics. Means, standard deviations, and comparison across the treatment groups. *p*-values are based on t-tests, except for sex, which is based on chi-square tests.

	AT		EX		
	Mean	SD	Mean	SD	*p*-Value
Sex (F/M)	4/4		5/3		0.61
NPQ at baseline (%)	34.7	11.7	29.9	7.4	0.34
Sedentary (hrs/day)	6.1	4.6	6.9	2.6	0.70
Age (yrs)	49.3	11.0	54.8	18.9	0.49
Education (yrs past HS)	5.3	3.4	7.5	3.3	0.21
Classes (# attended)	9.0	0.0	8.4	2.3	0.46

AT = Alexander technique. EX = exercise.

**Table 2.** Average rating for survey responses at second post-test.

	AT	EX	*p*
1. The classes were enjoyable.	9.1	8.3	
2. The material was presented in a clear and understandable way.	8.3	9.0	
**3. I learned about how my movements contribute to my neck pain.**	8.4	5.4	0.03
**4. I was surprised by some of the things I learned.**	9.0	4.1	0.001
5. I learned some practical tools to be more comfortable in my body.	8.5	6.6	
6. I am likely to remember what I learned.	8.0	7.1	
7. I am likely to continue to practice what I learned.	7.8	6.0	
8. I enjoyed the interaction with my fellow-students.	8.9	8.2	
9. I would refer a friend to this class.	8.8	6.9	
10. I would pay for continuing classes if they were available.	6.1	4.0	
11. I would have preferred a private lesson format.	1.9	3.6	
12. I would have preferred a class that met only once per week.	3.0	3.9	
13. I would have preferred a class that met more than twice per week.	1.0	3.1	
14. I would have preferred to meet in a more “healing” environment such as a yoga studio.	3.4	3.1	
15. I would have preferred a class with a more structured format.	1.5	2.8	

Rating scale (0–10): 0 (not at all/definitely not) to 10 (quite a lot/definitely yes). Rows with a p-value are significantly different between groups based on a 2-tailed t-test. AT = Alexander technique. EX = exercise.

**Table 3.** Survey responses at second post-test.

		Never or A Few Times	Weekly or Every Few Days	Daily or More Often	*p*
**I apply what I learned in the class to an activity we practiced in class.**	AT		xx	xxxxxx	0.007
	EX	xx	xxxxxx		
I apply what I learned in the class to an activity that we did NOT practice in class.	AT		xxxxxx	xx	
	EX	xxxx	x	xxx	
**I notice myself using my usual way of doing something and choose a different way.**	AT		xxx	xxxxx	0.006
	EX	xxx	xxxx	x	

Rows with a p-value are significantly different between groups according to a 2-tailed Mann-Whitney test. AT = Alexander technique. EX = exercise.

**Table 4.** Correlations between neck pain and possible predictor variables.

Predictor	Correlation with NPQ at Baseline	Correlation with NPQ at Post-Test	Correlation of Change in NPQ with Change in Predictor
Pain self-efficacy	0.04 (0.40)	P1: −0.38 (0.07) **P2: −0.76 (0.0003)**	P1-B1: 0.08 (0.40) **P2-B1: −0.39 (0.14)**
Head-torso angle	0.06 (0.08)	P1:0.23 (0.39) P2: 0.15 (0.58)	P1-B1: −0.17 (0.58) P2-B1: −0.05 (0.85)
Head angle	−0.36 (0.17)	**P1: −0.39 (0.14)** P2: 0.07 (0.80)	P1-B1: 0.03 (0.90) P2-B1: 0.03 (0.90)
Neck angle	0.13 (0.63)	P1: 0.25 (0.35) P2: −0.12 (0.65)	P1-B1: −0.04 (0.90) P2-B1: −0.08 (0.77)
Muscle amplitude	−0.04 (0.88)	P1: 0.23 (0.41) **P2: 0.65 (0.009)**	P1-B1: −0.08 (0.77) P2-B1: 0.19 (0.48)
Muscle frequency	−0.10 (0.72)	P1: 0.19 (0.50) P2: 0.21 (0.45)	P1-B1: 0.28 (0.31) P2-B1: 0.12 (0.67)

## Data Availability

Data are available upon request.
